# Treatment for Viral Hepatitis as Secondary Prevention for Hepatocellular Carcinoma

**DOI:** 10.3390/cells10113091

**Published:** 2021-11-09

**Authors:** Saleh A. Alqahtani, Massimo Colombo

**Affiliations:** 1 Division of Gastroenterology and Hepatology, Johns Hopkins University, Baltimore, MD 21287, USA; 2 Liver Transplant Center, and Biostatistics, Epidemiology, and Scientific Computing Department, King Faisal Specialist Hospital & Research Center, Riyadh 11564, Saudi Arabia; 3 Liver Center, IRCCS San Raffaele Hospital, 20132 Milan, Italy; mcolombo46@yahoo.it

**Keywords:** HCC, HBC, HCV, hepatocellular carcinoma, prevention

## Abstract

Chronic infections with either hepatitis B or C virus (HBV or HCV) are among the most common risk factors for developing hepatocellular carcinoma (HCC). The hepatocarcinogenic potential of these viruses is mediated through a wide range of mechanisms, including the induction of chronic inflammation and oxidative stress and the deregulation of cellular pathways by viral proteins. Over the last decade, effective anti-viral agents have made sustained viral suppression or cure a feasible treatment objective for most chronic HBV/HCV patients. Given the tumorigenic potential of HBV/HCV, it is no surprise that obtaining sustained viral suppression or eradication proves to be effective in preventing HCC. This review summarizes the mechanisms by which HCV and HBV exert their hepatocarcinogenic activity and describes in detail the efficacy of anti-HBV and anti-HCV therapies in terms of HCC prevention. Although these treatments significantly reduce the risk for HCC in patients with chronic viral hepatitis, this risk is not eliminated. Therefore, we evaluate potential strategies to improve these outcomes further and address some of the remaining controversies.

## 1. Introduction

Primary liver cancer ranks as the sixth most common cancer globally and the third leading cause of cancer-related death [1]. Hepatocellular carcinoma (HCC) is by far the most dominant histological subtype of liver cancer, accounting for about four-fifths of all cases [2]. Globally, the incidence of HCC is increasing, with GLOBOCAN predicting an increase from 841,000 cases in 2018 to 1.4 million cases in 2040. In parallel, it also predicts increased mortality, from 780,000 deaths in 2018 to 1.3 million in 2040 [3]. Infections with hepatitis B (HBV) or C virus (HCV) represent the most significant risk factors for the development of HCC, followed by heavy alcohol consumption. Epidemiological data from 50 countries indicate that at least 60% of all HCC cases are attributable to either HBV or HCV [4]. Although virus-related HCC is prevalent worldwide, there are notable geographical variations in the proportion of patients with HCC associated with HCV versus those with HBV. Reflecting the incidence and distribution of these two hepatitis viruses, HBV-induced HCC is more common in countries with a low to middle human development index. In contrast, HCV is responsible for most virus-related HCC cases in regions with a higher developmental index [5].

## 2. HBV

HBV is a small, partially double-stranded DNA virus belonging to the hepadnaviridae family. In 2015, 257 million persons, 3.5% of the global population, were living with a chronic HBV infection, making it the most common blood-borne infection in the world [6]. Patients with chronic hepatitis B (CHB) may develop cirrhosis, and the 5-year cumulative incidence ranges between 8% and 20%. About 2% to 5% of patients who develop cirrhosis may then face an HCC risk annually [7]. During early 1980s, a hepatitis B vaccine was developed, successfully reducing the disease burden. By 2015, the global coverage of persons with three doses of this HBV vaccine was 84%. Consequently, the global proportion of HBV-infected children younger than five years old dropped from 4.7% in the pre-vaccination era to 1.3% in 2015 [8]. Evidence continues to accumulate, showing that this mass vaccination substantially reduces the disease burden associated with HBV. For example, mass vaccination campaigns in China reduced the prevalence of new HBV infections by 90%. Models estimate that this prevented around 2.8 million to 3.5 million deaths associated with HBV in the future. Most were predicted to be associated with HCC [9]. For chronic HBV carriers, the current therapeutic options do not provide a virological cure, but treatments with nucleos(t)ide analogs (NAs) can effectively reduce the risk of HCC [10,11].

## 3. HCV

HCV is a single-stranded RNA virus belonging to the Flaviviridae family. Every year, approximately 1.75 million persons are infected with HCV, and HCV-related liver cirrhosis or HCC results in the death of over 350,000 patients. In 2020, 58 million people were estimated to have chronic HCV infection [8]. In patients with a chronic HCV infection, the risk of HCC gradually increases with the progression of liver fibrosis. Once cirrhosis is established, the annual incidence of HCC is high at 2 to 8% per year [12]. In contrast to HBV, there is currently no preventive vaccine for HCV. However, over the last decade, very effective direct-acting anti-viral agents (DAAs) have been developed that can cure the vast majority (>90%) of HCV patients. Studies with these agents have convincingly demonstrated that obtaining a sustained virological response (SVR) with DAAs significantly reduces the risk of HCC [13].

To underscore the importance of viral clearance in the prevention of HBV- or HCV-related HCC, this review provides firstly a brief overview of the molecular mechanisms that form the basis of the oncogenic potential of these viruses. Subsequently, we provide evidence supporting the efficacy of the current HBV and HCV treatment strategies in averting the development of HCC. With these treatment modalities, the risk for HCC in patients with chronic viral hepatitis is significantly reduced but not abolished. Therefore, we address potential strategies to improve these outcomes further and discuss some of the remaining controversies.

## 4. Molecular Mechanisms by Which HBV and HCV Induce HCC

**Figure 1:**
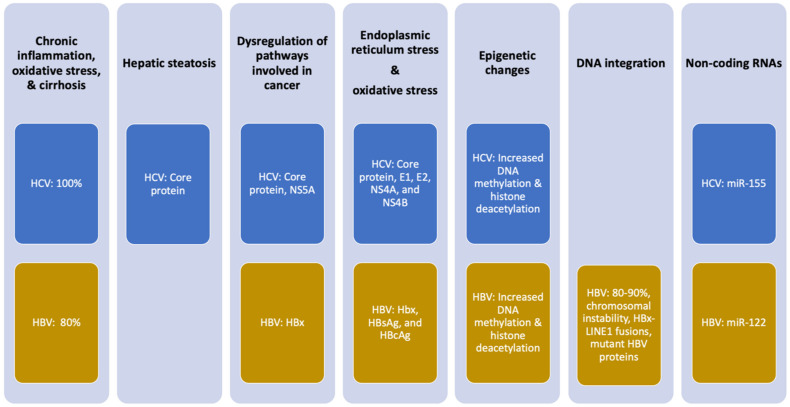
Mechanisms of oncogenesis by HCV and HBV. Percentages indicate the frequency with which the effect is observed in patients with infection-associated HCC. Viral proteins implicated in the oncogenic mechanism are noted.

Chronic infections with HBV or HCV significantly increase the risk of developing HCC [7,14,15]. However, not all persistently infected individuals develop liver cancer, indicating that chronic infection with these viruses by themselves is generally not enough to develop cancer. As with many cancers, multiple factors contribute to oncogenesis. HBV and HCV only provide a portion of the oncogenic pressure (Figure 1) and combine with other factors (such as environmental, lifestyle, or genetic factors) to develop HCC [16].

## 5. Chronic Inflammation

A crucial mechanism by which chronic HCV and HBV drive the development of HCC involves chronic liver inflammation. HCV-related HCC often develops exclusively in the context of liver cirrhosis [17]. Although most HCC cases develop in patients with chronic HBV infection and cirrhosis, there are still 10–20% of HBV-related HCC cases that occur in the absence of cirrhosis [18]. The persistence of inflammatory stimuli resulting from a chronic HCV or HBV infection combined with dysregulated immune regulatory mechanisms causes a state of constant inflammation, leading to fibrosis, cirrhosis, and neoplastic transformation in the liver parenchyma. This immune-mediated liver damage is mainly caused by the release of reactive oxygen species (ROS) and pro-inflammatory cytokines by hepatocytes and immune cells (in particular, natural killer cells and T cells). The resulting necroinflammation stimulates hepatocyte regeneration and wound healing. When a patient fails to clear HBV or HCV completely, this constant process of necroinflammation and subsequent wound healing responses gradually increases the oxidative stress in the liver, leading to the induction of epigenetic and oncogenic alterations, telomere shortening, and genomic instability [19]. Specifically for HCV, researchers have found that the incomplete eradication of HCV results in the selection of escape mutants that evade the immune system and establish a chronic infection [20]. In addition to fueling the persistent inflammatory state, the release of ROS and specific cytokines results in the activation of stellate cells and fibroblasts in the liver. In turn, these cells enhance the synthesis of collagen and alter the extracellular matrix, ultimately leading to fibrotic remodeling of the liver microenvironment [21].

## 6. Viral Proteins Deregulate Cellular Pathways and Induce Oxidative Stress

In addition to the inflammation-mediated, indirect oncogenic effect, both HCV and HBV also directly influence hepatocarcinogenesis. This direct oncogenic effect involves specific viral proteins.

The HBV protein HBx exhibits carcinogenic activity. HBx modulates several inflammation pathways such as signal transduction and, consequently, the progression of liver disease, including the MAPK, NF-κB, IL-6/STAT3, and PI3K pathways, as well as signaling by Src [22]. HBx also activates the Wnt/β-catenin pathway, leading to an accumulation of β-catenin and the subsequent induction of pro-angiogenic factors [23]. Interestingly, HBx binds to the tumor suppressor p53 in the cytoplasm, preventing the translocation of p53 into the nucleus, where p53 is involved in responding to DNA damage by activating cell cycle checkpoints, apoptosis, and DNA repair [24]. Ultimately, this hampered p53 activity leads to genomic instability, which drives neoplastic transformation. A second mechanism by which HBx promotes the development of HCC consists of epigenetic regulation. HBx encourages the expression of several DNA methyltransferases, which in turn regulate the face of a broad range of proto-oncogenes and tumor suppressors via hypo- and hypermethylation. HBx also exhibits epigenetic activity through the promotion of histone (de)acetylation of cancer-related genes, microRNAs (e.g., miR-122), and non-coding RNAs [25].

In HCV-infected hepatocytes, the HCV core protein and NS5A are essential for altering signaling through pathways involved in cancer [26]. The HCV core protein impacts cell growth, differentiation, apoptosis, transcription, and angiogenesis. Its mechanism of action is to activate MAPK, Wnt/β-catenin, TGF-β, PI3K/Akt/mTOR, NF-κB, IL-6/STAT3, and androgen receptor signaling and suppress apoptotic signaling. Similarly, NS5A engages with several pro-oncogenic pathways, such as β-catenin, PI3K/AKT/MTOR, NF-κB, and p53 [27].

Both viruses influence chromatin structure and gene expression by altering epigenetic regulation and the production of microRNAs. Through these mechanisms, viral proteins from each virus also impact pathways implicated in oncogenesis and contribute to HCC development. HBx of HBV and HCV core protein alter epigenetic events by increasing DNA methyltransferase activity and histone deacetylation [28,29]. HBx also increases miR-122 and other non-coding RNAs [25]. HCV core protein increases miR-155, which is markedly increased in patients infected with HCV and stimulates hepatocyte proliferation and tumorigenesis by activating Wnt signaling [30,31].

Both HBV and HCV replication involves the endoplasmic reticulum, the site where viral proteins are produced, and the source of the lipids surrounding the virus. Along with HBx, the HBV proteins HBsAg and HBcAg are associated with the induction of oxidative stress as a result of impaired protein folding in the endoplasmic reticulum. The HCV core, E1, E2, NS4A, and NS4B proteins also cause this endoplasmic reticulum stress [19]. During infection, the production of these viral proteins leads to the accumulation of misfolded proteins in the endoplasmic reticulum, which activates the unfolded protein response. As part of this response, calcium ions are released from the endoplasmic reticulum into the cytoplasm, which stimulates ROS production that can induce inflammation, tissue damage, and fibrosis and contribute to the development of HCC [19].

## 7. HCV-Induced Hepatic Steatosis

A specific oncogenic effect of HCV relates to hepatic steatosis, which is defined as an excessive triglyceride presence in hepatocytes. Hepatic steatosis is independently associated with HCC development in patients with HCV-related cirrhosis [32]. HCV core protein alters lipid metabolism. In a transgenic mouse model, overexpression of HCV core protein reduces triglyceride transfer protein activity, which results in the accumulation of triglycerides in hepatocytes and causes oxidative stress that contributes to an oncogenic liver environment [33].

## 8. HBV Integration in Host DNA

A carcinogenic mechanism specific to HBV is the genomic integration of HBV DNA into the hepatocyte genome, a feature that is observed in 80–90% of HBV-related HCC cases [34]. HBV DNA integration appears to occur early in the infection. Viral DNA integration fuels hepatocarcinogenesis through three mechanisms. First, HBV DNA integration causes genomic instability, a known hallmark of cancer. Although integration occurs in most infected individuals, those without HCC have HBV sequences randomly integrated throughout the genome. In contrast, HBV DNA integration in patients with HCC is enriched in regions known to cause chromosomal instability [35]. Secondly, more comprehensive sequencing studies show recurrent HBV DNA integration sites at genetic loci that encode for proteins with significant involvement in initiating HCC carcinogenesis [34]. A prime example is HBx-LINE1 gene fusions, a genomic aberration that can be found in about a quarter of HBV-associated HCCs [36]. Finally, the integration of HBV DNA can also lead to the production of mutant HBV proteins, leading to an overload of the endoplasmic reticulum, resulting in oxidative stress [37].

## 9. Predictors of HCC and Screening Strategies

The neoplastic transformation of chronic hepatitis B is driven by an interplay between direct and indirect mechanisms of carcinogenesis in the context of chronic inflammation and the regeneration of liver cells [26]. Understanding factors that at the individual level promote liver carcinogenesis is of paramount importance for the configuration of cost-effective screening programs and linkage to patient care. Not surprisingly, several viral covariates have been associated with HBV transition to HCC, the most robust predictor of HCC being a high viral load, independent of the patient’s cirrhosis status or high HBsAg levels [38,39,40]. Thus, this finding sets the stage for anti-viral therapy to become the only realistic approach to temper the risk of the neoplastic transformation of chronic HBV infection progression.

The follow-up reanalysis of 48,149 patient years from the REVEAL study in Taiwan offered essential insights into the association between the lifetime risk of HCC and the HBV infection phase. HCC risk was strikingly high in males and HBeAg seroconverters who had remained HBV DNA-seropositive compared to those who cleared both HBsAg and HBV DNA (80.1% vs. 4.0%) [41]. However, HBsAg seroclearance occurs in less than 10% of patients treated with NAs and interferon (IFN) and is associated with HBV DNA detectability in the liver [42,43]. The HCC risk is higher in older HBV patients, implying the pathogenic relevance of the infection length and the age-dependent loss of protective mechanisms against cancer. In a multiethnic milieu such as the US, the HCC risk was higher in HBV carriers of African American origin than in Whites, Latinos, and Asians [44]. Together with genetic studies, these findings highlighted the importance of genetics as an outcome modifier of HBV infection and opened the way to studies of single-nucleotide polymorphisms that have been identified to confer susceptibility to HCC. In contrast, evidence started to accumulate that an interaction between a genetic predisposition to HCC and the lifestyle choices of HBV carriers have a severe impact on the risk of HCC development. One study demonstrated the lethal consequences of alcohol abuse that have long been recognized in both hemispheres, since drinking alcohol accelerates the development and progression of liver cirrhosis and HCC in HBV patients in a dose-response pattern, and some studies also exhibit a positive interaction with tobacco smoking [45]. In the seminal studies performed decades ago, tobacco was found to boost the risk of HCC development in HBV patients independently of other confounders, including alcohol. This association was later confirmed by a large meta-analysis, in which the hazard (random effect) of HCC development was 1.87, 15.8, and 21.6 for HBV-seronegative smokers, HBV-seropositive non-smokers, and HBV-seropositive smokers, respectively [46]. In HBV-infected patients, steatosis is related to an increased risk of liver fibrosis, followed by HCC progression, independently of anti-viral therapy [47,48]. In a meta-analysis including almost 22,000 patients, the presence of diabetes, a dominant culprit in liver steatosis, was associated with significantly increased overall mortality (pooled RR: 1.93 (1.64–2.27)) compared to HBV carriers without diabetes [49].

## 10. Prediction Models and Scoring Systems for HCC Development

Ultimately, the essential variables concerning the HCC risk in hepatitis B carriers are linked to the liver disease stage and hepatic fibrosis. The stage of liver fibrosis can be assessed with non-invasive methods that have been validated in patients with other chronic liver diseases. The most popular method includes transient elastography, which measures liver stiffness. Scores based on a combination of serum markers [50,51,52], which incorporate host, viral, and liver disease variables, accurately predict the HCC risk in patients with a chronic HBV infection. (Table 1) [40,47,53,54,55]. Following the selection of an appropriate cut-off value, all these scores for HCC exhibit a substantial negative predictive value in the range of 5 to 10 years. The European Association for the Study of the Liver (EASL) endorses the PAGE-B score for HCC risk stratification, recommending biannual screening with abdominal ultrasound for HBV carriers with a score of 10 or more [56]. Full descriptions of each scoring system are beyond the scope of this review but are summarized in Table 1.

## 11. Anti-HBV Therapy as Secondary HCC Prevention

An initiative to increase HBV awareness and treatment was commenced by the WHO and other health agencies in 2016, with the aim of eliminating viral hepatitis by 2030 [58]. High-impact interventions were planned, followed by studies that modeled the hepatitis epidemiology and covered aspects such as increasing sanitation, newborn mass vaccination against HBV, screening, and linkage to care of the infected populations. In patients with an untreated HBV infection, the incidence of HCC increases with the increase in the serum HBV DNA level. The advised first-line regimen for chronic hepatitis B is INF-α and tenofovir (TDF) and entecavir (ETV), which reduces hepatic inflammation via viral suppression [56]. Randomized controlled trials and meta-analyses have demonstrated that administering IFN for a finite duration reduces the risk of HCC in treated compared to untreated patients [59,60,61,62]. In most patients, the continued administration of either NA has caused attenuation, but unfortunately not the eradication, of the HCC risk. The persistence of the HCC risk results from NA failing to eradicate cccDNA and the integrated sequences of the HBV DNA. This leads to the limited rates of serum HBsAg clearance in NA-treated patients, whereas the persistence of residual HCC risk has been recognized even in patients who do not show serum HBsAg following anti-viral therapy, specifically in those patients with HBsAg seroclearance occurring after 50 years of age [56]. As per a joint report of the American and European Societies of the Study of the Liver, the pragmatic goal of NA therapy is to achieve a functional HBV cure, defined as permanent HBsAg clearance with or without HBsAg seroconversion after treatment completion [63]. In real-world practice, however, durable suppression of serum HBV DNA coexisting with detectable HBsAg is the expected outcome of most patients treated with NA [11,55,64].

Several large-scale epidemiological studies identify high levels of HBV DNA in the serum as a critical risk factor for HCC development in chronic HBV cases [65,66]. The REVEAL-HBV study, which followed more than 3600 HBsAg carriers for an average of 11 years, revealed an independent dose-dependent relationship between a serum HBV DNA level above 2000 IU/mL and HCC development [39]. Other HBV-related factors associated with an increased risk for HCC include specific variations in the HBV DNA sequence, the HBV genotype, mutations in the basal core promoter, and levels of quantitative HBsAg [67,68,69]. Given the close relationship between these viral characteristics and the risk for HCC, HBV replication is a logical target for preventing HCC in this setting.

The first evidence for preventing HCC with anti-viral therapy in patients with chronic HBV comes from studies with older NAs, such as lamivudine and adefovir. Liaw et al. reported that lamivudine (with a mean treatment duration of 3 years) significantly reduced the HCC risk compared to a placebo (HCC incidence 3.9% vs. 7.4%; hazard ratio (HR): 0.49; *p* = 0.047) [70]. A meta-analysis, including more than 6800 patients, reported similar 4-year HCC rates in lamivudine-treated and control patients [71]. However, these first-generation NAs are no longer used, mainly due to suboptimal virological responses associated with HBV resistance, which is referred to as a low genetic resistance barrier. Currently, the treatment guidelines for HBV unanimously recommend the use of ETV or TDF [56,72]. These agents are well-tolerated and have a much higher genetic resistance barrier. Their long-term use induces sustained virological suppression in the vast majority of patients (>95%), along with histological improvements and regression of fibrosis and cirrhosis [73,74]. Furthermore, cohort and population studies carried out all across the world demonstrate that long-term use (>5 years) of either ETV or TDF prevents the development of HCC in the majority of patients [11,75,76,77]. A cohort study from Hong Kong including 1225 chronic HBV patients who were treated with ETV between 2002 and 2015 compared the HCC incidence among ETV-treated patients to the expected HCC incidence calculated with well-established HCC risk scores for a patient with chronic HBV [76]. The reduction in HCC risk achieved with ETV was significant starting from year six of treatment with a standardized incidence ratio of 0.68 (95% CI: 0.535–0.866). Notably, the HCC-preventing effect of ETV was seen in both cirrhotic and non-cirrhotic patients [76].

## 12. Tenofovir or Entecavir: One Better Than the Other?

Current international treatment guidelines recommend ETV or TDF as equal first-line treatment options for chronic HBV [56,72]. However, several non-randomized studies suggest a lower HCC incidence with TDF than with ETV, which has fueled a discussion of whether one NA is superior to the other. This discussion was kicked off by the results of a large Korean population study indicating a significantly lower risk for HCC in chronic HBV patients treated with TDF compared to those treated with ETV (HR: 0.68) [78]. However, a second Korean cohort study with a largely overlapping patient cohort failed to confirm this and did not show any difference in HCC risk between ETV and TDF (HR: 1.02) [79]. Subsequently, several other observational studies and meta-analyses did not conclude and support the superiority of a single regimen compared to the other [80,81,82,83,84]. The systematic review and meta-analysis by Tseng et al. may resolve this controversy [85]. This analysis corrected some important limitations of earlier attempts to address this point. Confounding factors that were appropriately resolved included addressing the heterogeneous populations, including only a few comparative studies, the pooling of unadjusted data with adjusted data, the presence of overlapping cohorts, and the analysis of HCC data as a dichotomous outcome instead of through time-to-event data. Tseng et al. included 31 studies representing 119,053 patients. When considering the eight studies with matched populations using propensity score matching, there was no difference in the pooled 5-year cumulative HCC incidence between the two NAs (*p* = 0.87). However, elastographic cirrhosis reversion (liver stiffness <12 kPa) after five years of treatment was seen in 71% of patients with pretreatment liver cirrhosis, and it was more common in TDF- than in ETV-treated cases (74% vs. 62%, *p* = 0.04) [86]. Another meta-analysis analyzing 13 studies with multivariable or propensity-score-matched risk assessments, involving around 4000 HCC cases and 80,000 HBV HCC-free patients, showed no difference in HCC incidence between the TDF and ETV treatment groups (HR 0.86, 95% CI 0.72–1.04) [87]. This outcome was consistent with the propensity-score-matched meta-analysis (HR 0.83, 95% CI interval 0.66–1.03) and the subgroup analysis, revealing the marked resemblance of TDF to ETV for HCC prevention, continued in studies with a longer follow-up of more than four years. However, the finding that the similarity between TDF and ETV for HCC prevention was lost among patients with a follow-up time shorter than four years (*p* interaction < 0.01) led the authors to speculate that the heterogeneous effects of included reports might emerge from the variation in the follow-up time. As all the above-discussed meta-analyses were draining data from the same pool of studies, it is worth noting that more robust scrutiny through single-data patient meta-analysis or a large-scale randomized comparative trial might help to solve the controversy as to which regimen dominates the chemoprevention of HBV-related HCC.

## 13. Can We Prevent More Cases of HCC by Treating HBV Patients Earlier?

The most recent guidelines for HBV treatment (EASL/AASLD) recommend that anti-HBV treatment in people aged ≥30/40 years with a viral load of >2000 IU/mL is indicated even in the absence of significant fibrosis or increased alanine aminotransferase (ALT) [56,72].

**Figure 2:**
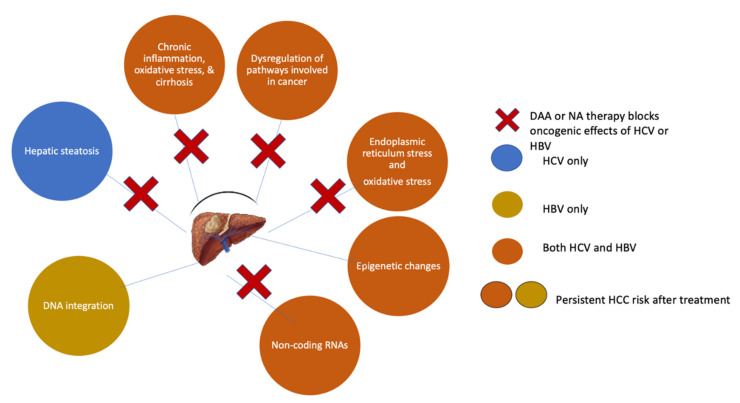
Mechanisms of HCC prevention and risk persistence following DAA or NA therapy.

However, abiding by these recommendations does not completely eradicate the risk of HCC (Figure 2). Long-term follow-up data demonstrate a low but persistent risk for HCC in patients treated with long-term NA therapy (annual risk 0.5–1.4% in non-cirrhotic patients and 0.7–5.0% in cirrhotic patients) [10]. In addition, studies indicate that the incidence of HCC only starts to drop after five years of NA therapy [10,11,88]. Thus, an extended treatment duration might be necessary to maximize the preventive HCC effect and fuel a discussion of whether starting treatment earlier than currently recommended might improve clinical outcomes and decrease the HCC risk [11]. Retrospective data reported by Kim et al. showed that chronic HBV patients who remained untreated had a 2- to 3-fold increased risk of HCC development, requiring liver transplantation, or dying than patients who initiated NA therapy [89].

Initiating anti-viral therapy at an earlier HBV disease stage also has a mechanistic rationale. Studies indicate that genomic integration of HBV DNA, which is a critical oncogenic driver of HBV-related HCC, is frequently detected in young patients with immune-tolerant disease [90]. Additionally, other pro-oncogenic features of HBV, including HBV-specific T cell responses and hepatic necroinflammatory activity, are often detectable in young immune-tolerant patients [91]. Based on these findings, it is tempting to assume that hepatocarcinogenesis starts in the immune-tolerant phase of chronic HBV and that oncogenic events accumulate throughout the disease. Thus, the current treatment paradigms would enable decades of unhindered oncogenic events, which a “treatment-for-all” approach would prevent.

Apart from potentially preventing additional cases of HCC, adopting a treatment-for-all approach in which younger chronic HBV patients are treated with NAs would yield other advantages. It would prevent further patient transmission of HBV and would simplify the management of chronic HBV patients. It would shift the paradigm from a model necessitating multiple tests to assess treatment eligibility (serology, assessment of HBV DNA level, evaluation of liver fibrosis, etc.) to a simple “test-and-treat” approach. Moreover, in the current era of generic NAs, the costs of NA treatment have become lower than the costs of disease monitoring. Cost-efficacy analysis shows that initiating anti-viral therapy in the immune-tolerant phase is cost-effective compared to delaying therapy until patients have progressed to the active hepatitis phase [92]. However, hurdles are still in place preventing the implementation of such a treatment-for-all approach. First, models estimate that nine out of ten chronic HBV patients are not aware that they had the infection, and even when detected, subsequent linkage to care with NA therapy remains suboptimal [93]. A second hurdle relates to the need for lifelong (or prolonged) treatment to prevent a flare-up of the infection, but such a prolonged treatment regimen comes with treatment compliance issues.

## 14. Is There a Role for Statins and Aspirin?

Both aspirin and statins can affect liver cell metabolism and the inflammation associated with hepatocarcinogenesis. A large-cohort study, including over 7700 patients with chronic HBV followed for a median of 7.2 years, showed that statin use (defined as 28 cumulative daily doses) was associated with a 64% reduced risk for HCC compared to no statin use (adjusted HR (95% CI): 0.36 (0.19–0.68)). This HCC prevention effect of statins was dose-dependent and observed in all investigated subgroups [94]. The proposed mechanisms of this anticancer effect, which is not limited to HCC but has been reported for other cancers, are believed to be mediated by inhibiting the mevalonate pathway and its downstream products. These products are critical for malignant cell proliferation and the inhibition of hepatic fibrogenesis [95,96,97]. In addition to this, statins may slow down the synthetic process of cholesterol and the replication of HBV, thus possessing anti-HBV activity [98].

A similar preventive HCC effect for aspirin was described in a cohort study conducted across Taiwan, including over 10,000 patients with chronic HBV. The cumulative incidence of HCC was significantly lower among chronic HBV patients who received daily aspirin for ≥90 days (*n* = 2123) compared to patients who did not (*n* = 8492) (5.2% vs. 7.87%; HR (95%C): 0.71 (0.58–0.86); *p* < 0.001) [99].

Recently, Simon and colleagues analyzed the effect of aspirin on HCC development in 14,205 chronic HBV or HCV patients, with low-dose aspirin intake and found after 7.9 years of follow-up, aspirin significantly lowered the rate of HCC compared to 50,275 chronic HBV or HCV patients with no aspirin intake (4.0% vs. 8.3%; HR (95%C): 0.69 (0.62–0.76); *p* < 0.001) [100].

## 15. Novel Treatments

Hepatic neoplastic transformation in patients who carry HBV involves the clearing process of nuclei (present in infected hepatocytes) from HBV DNA sequences that are integrated into chromosomes and also from free viral cccDNA. Unfortunately, NAs fail to clear those. This may partly explain the residual risk for HCC seen in chronic HBV patients treated with these agents. NA adequately controls HBV replication, but a cure is rare. Therefore, therapy has to be given indefinitely, raising the prospect of selecting drug-resistant virus variants. Pegylated-IFN-α-based therapies may in some cases cure the HBV infection but suffer from a moderate response rate and severe side effects. Advances in understanding the HBV life cycle are being exploited to develop novel anti-viral agents that suppress HBV replication and inhibit the formation of cccDNA. As the persistence of cccDNA characterizes chronic hepatitis B, this could be a potential target, which is currently not targeted by approved drugs. Agents under investigation include those that block HBV entry into hepatocytes, target cccDNA using CRISPR technology or epigenetic silencing, promote the degradation of viral RNA with RNA interference molecules, or disrupt the production and secretion of viral proteins. Strategies are also used to restore or enhance the anti-HBV immune response.

Moreover, targeting host factors contributing to the life cycle of HBV may present new possibilities for developing innovative therapeutic strategies aiming at an HBV cure. Recent advances in understanding HBV–host interactions highlight how exploiting host-targeting may lead to a viral cure strategy. Although some of these agents have yielded promising results in early clinical studies, the holy grail of a functional cure for HBV remains elusive [101,102].

## 16. Anti-HCV Therapy for the Prevention of HCC

Like HBV, active HCV infection is central to the hepatocarcinogenic process. Thus, viral elimination is the goal for the secondary prevention of HCC. In this respect, studies from the IFN era convincingly demonstrated that an SVR following IFN-based therapy reduced the risk for HCC to 0.5–1% per year (vs. 2–8% per year in untreated chronic HCV patients with cirrhosis) [12,103]. Unfortunately, an SVR to IFN-based therapy was only achieved in half of the patients. Furthermore, IFN toxicity limits its use in patients with cirrhosis.

During the last decade, IFN-free DAA drugs have revolutionized the HCV treatment landscape. An SVR is obtained with these agents in >95% of patients, and most show improvements in liver fibrosis and liver function and a reduction in portal hypertension [104]. Moreover, these agents have an excellent safety profile and minimum adverse effects and can be used in patients with decompensated liver disease. Achieving SVR is proven to be beneficial at all stages of fibrosis, including in patients with decompensated cirrhosis; however, the elimination of risk in HCC in patients with decompensated cirrhosis cannot be achieved; therefore, surveillance for HCC is extremely important for patients with advanced fibrosis or cirrhosis after achieving SVR. [105]. Bruno et al. discuss the ‘point of no return’, since disturbances to the liver architecture in decompensated cirrhosis tend to have a poor prognosis that leads to the development of HCC [106]. Box 1 provides the summary of treatment options for viral hepatitis that are under consideration or development aimed towards HCC prevention.

Box 1 **Summary of viral hepatitis treatment options under current consideration or development aimed towards HCC prevention.**

•  Current international treatment guidelines recommend ETV or TDF as equal first-line treatment options for chronic HBV.

•  Pegylated-IFN-α-based therapies in selected patients may cure HBV infection; however, it can be associated with significant severe side effects.

•  Novel agents under investigation include those that block HBV entry into hepatocytes, target cccDNA using CRISPR technology or epigenetic silencing, promote the degradation of viral RNA with RNA interference molecules, or disrupt the production and secretion of viral proteins.

•  Recent advances in understanding HBV–host interactions highlight how exploiting host-targeting may lead to a viral cure strategy.

## 17. HCC Occurrence after DAA Therapy

Given the high rate of SVR obtained with DAA, it decreases the risk of HCC and thus reduces the HCV-related morbidity and mortality rates. However, two early reports suggested an unexpectedly high incidence of HCC in HCV patients who achieved SVR after DAA therapy (a 1-year cumulative HCC rate of 3.6% in one study, a 6-month HCC rate of 4% in the other) [107,108]. Cardoso et al. subsequently reported a 1-year HCC rate of 7.4% and a short median time to HCC development of 7 months [109]. In a study by Ravi et al., an even higher 6-month HCC incidence of 9% was reported in a cohort of 66 chronic HCV patients with cirrhosis [110]. These controversial results sparked a heated debate on the risk of HCC following an SVR on DAA therapy and fueled hypotheses on the potential involvement of DAAs in hepatocarcinogenesis. However, these initial studies had some crucial drawbacks. They were all characterized by a small sample size, a short follow-up period, and the absence of a control arm. As such, it is no surprise to see that a stream of subsequent studies, both retrospective and prospective, counteracted these findings and firmly established the HCC chemopreventive potential of DAA therapy (Table 2).

A retrospective study by Singer et al. compared the HCC incidence between untreated chronic HCV patients (*n* = 137,502) and patients treated with DAA (*n* = 30,183) or IFN-based therapy (*n* = 12,948). After adjusting for age and cirrhosis status at baseline, DAA-treated patients had a significantly lower risk of HCC than untreated patients (HR (95% CI): 0.84 (0.73–0.96)) and then patients treated with IFN (HR (95% CI): 0.69 (059–0.81)) [111]. A similar 70% HCC risk reduction with DAA therapy vs. no treatment was reported by Janjua et al. [112].

The importance of adjusting for confounding factors is illustrated by a final retrospective study by Nahon et al., which showed a higher 3-year HCC incidence in patients treated with DAAs compared to those obtaining an SVR with IFN (5.9% vs. 3.1%; unadjusted HR (95% CI): 2.03 (1.07–3.84); *p* = 0.030) [113]. However, it is essential to underscore that this study did not adjust for differences in patients and disease characteristics between the DAA and IFN cohorts. This is particularly important because IFN-based therapy was mainly used in younger chronic HCV patients with mild to severe fibrosis. In contrast, the patients who received DAA therapy were predominantly those with cirrhosis and with advanced age. When an analysis does not correct for these differences, a higher HCC incidence in the DAA cohort is not surprising.

Several prospective studies further undercut the initial reports of a higher risk of early HCC with DAA therapy. A French study that included 7344 chronic HCV patients treated with DAAs and 2552 untreated patients confirmed that DAA treatment was associated with a significant decrease in HCC (adjusted HR (95% CI): 0.66 (0.46–0.91)) [114]. A prospective multicenter cohort study including 1400 Latin American patients with chronic HCV (median follow-up: 16 months) showed that an SVR with DAA regimens was associated with a 73% relative risk reduction for de novo HCC with a cumulative HCC incidence in cirrhotic patients of 0.02 and 0.04 at 12 and 24 months, respectively [115].

In conclusion, DAA therapy has a preventive effect on the development of HCC in patients with chronic HCV. The initial observation of an increased incidence of HCC in patients who achieved SVR after treatment with DAA therapy could be explained by the theory of early HCC development may have occurred in those with liver nodules undefined by magnetic resonance imaging while commencing DAA treatment. This may be promoted by the imbalance in field immunity caused by the swift eradication of HCV [126].

## 18. HCC Recurrence in DAA-Treated Patients

In the early DAA years, concerns were raised about a higher HCC recurrence risk in HCV patients who had HCC. The studies that formed the basis for the de novo HCC debate also reported an unexpectedly high rate of recurrent HCC, with an HCC recurrence in over a quarter of patients during the first six months following the start of the DAA treatment [107,108]. However, as for the de novo HCC risk, subsequent studies have tackled these initial observations and confirmed that DAA therapy reduces the risk of recurrent HCC compared to no treatment. A retrospective study by Lin et al. showed that DAA therapy improved the survival outcomes of HCC patients and did not increase recurrent HCC after curative therapy [127]. This conclusion was further reinforced by a large retrospective study from the United States and Canada, including 793 patients with HCV-related HCC, of whom 304 (38.3%) received DAA therapy and 489 (61.7%) remained untreated. Tumor recurrence was 42.1% in DAA-treated patients and 58.9% in untreated patients. As such, these findings underscore that DAA exposure is not associated with an increased risk of HCC recurrence (HR (95% CI): 0.90 (0.70–1.16)) [128]. Meta-analyses support this conclusion. A meta-analysis, including six studies with a follow-up of 1.35–4 years indicated a 64% lower risk for HCC recurrence in patients treated with DAA compared to controls (OR (95% CI): 0.36 (0.27–0.47); *p* < 0.001) [129]. Another meta-analysis, including a total of 2957 patients from 31 studies, found that DAA therapy reduces the risk of HCC recurrence compared to an IFN regimen (RR (95% CI): 0.64 (0.51–0.81)) and no intervention (RR (95% CI): 0.68 (0.49–0.94)) [130].

## 19. Residual HCC Risk Post-SVR and the Need for Surveillance

DAA therapy for HCV significantly reduces the risk for HCC but does not eliminate it (Figure 2). Although DAA therapy can improve advanced fibrosis and cirrhosis, this process takes time, during which patients still have an increased risk of HCC [131]. In some patients, SVR-induced regression of fibrosis or cirrhosis is counteracted by the acquisition of concomitant liver damage due to alcohol abuse or non-alcoholic steatohepatitis. Additionally, specific HCV-related genetic or epigenetic changes may persist in small pre-cancerous nodules even after achieving an SVR and predispose patients to HCC development. Indeed, Hamdane et al. found that epigenetic changes in histone H3K27ac in the liver tissue of untreated chronic HCV patients were present after HCV clearance by either DAAs or IFN [132]. Similarly, Perez et al. demonstrated persistent epigenetic alterations in liver biopsy samples from patients post-SVR and showed that this epigenetic signature could be reverted in vitro by drugs targeting a chromatin-modifying enzyme [133]. These epigenetic scars could not only act as potential early plasma biomarkers to identify patients at risk for HCC but might also represent therapeutic targets.

The ever-increasing number of patients with HCV clearance with DAAs, combined with the persistent risk of hepatocarcinogenesis even after SVR, emphasizes the need for tools that facilitate the identification of patients with the highest HCC risk. Such tools would enable the development of tailored surveillance programs. Several risk factors for HCC have been identified in patients with chronic HCV who obtained an SVR after anti-viral therapy. In most studies, older age and a fibrosis stage ≥F3/F4 were consistent significant risk factors for HCC [134]. In addition, a history of HCC, male sex, diabetes mellitus, heavy alcohol intake, and high gamma-glutamyl transferase and alpha-fetoprotein were found to be associated with a higher HCC risk post-SVR [116,135]. Basal liver stiffness has emerged as a potential predictive factor for HCC in this setting. Rinaldi et al. showed a significantly higher risk for patients with a liver stiffness of >30 kPa on FibroScan^®^ (HR (95% CI): 0.329 (0.131–0.830)). Degasperi et al. identified a baseline liver stiffness above 30 kPa as an independent predictor of de novo HCC (3-year HCC rate of 20% and 5% for patients with a liver stiffness >30 kPa and ≤30 kPa, respectively) [136].

This long list of risk factors underscores that liver histology is not the only predictor of HCC post-SVR and questions the current surveillance strategies that almost entirely depend on the stage of fibrosis [137,138]. It would be more accurate to estimate the HCC risk directly rather than indirectly by extrapolating from the fibrosis stage and use this HCC risk to decide the need for HCC surveillance. A simple tool to estimate the HCC risk in post-SVR HCV patients consists of the fibrosis-4 score (FIB-4), which is calculated using the circulating concentrations of aspartate aminotransferase (AST) and ALT, the age of the patient, and his/her platelet count. In post-SVR patients with HCV, a FIB-4 score of ≥3.25 identifies high-risk patients, and a score of <3.25 identifies low-risk patients [139]. Even non-cirrhotic patients with a FIB-4 score ≥3.25 maintain a considerable 1.22% annual HCC risk, warranting continued HCC surveillance. In contrast, the HCC risk in non-cirrhotic patients with an FIB-4 score below this threshold was very low at only 0.24%, indicating that these patients could potentially forego surveillance. Finally, a change in the FIB- 4 score before and after SVR also seems to hold predictive information. Patients with an FIB-4 score ≥3.25 before and after SVR had an exceptionally high HCC risk of approximately 2% per year. This was much lower in patients in whom the FIB-4 score dropped below 3.25 post-SVR [139]. Given the association between liver stiffness and HCC risk, FibroScan^®^-related liver stiffness could also be used to assess the HCC risk. Although easy to use, FIB-4 and liver stiffness assessments are not the most accurate tools to assess the HCC risk.

As a more precise alternative, several multivariable regression models have been developed. For example, data from the Veterans Affairs healthcare system was used to establish an HCC risk score that combines SVR, age, sex, body mass index, race or ethnicity, HCV genotype, platelet count, and the level of AST, ALT, albumin, INR, and hemoglobin to determine a patient’s HCC risk [140]. This model predicts that the presence of several HCC risk factors can result in an annual HCC risk of >1% in a proportion of non-cirrhotic post-SVR patients, indicating that such patients warrant more active surveillance. Another more straightforward risk score was recently developed (aMAP) that only relies on age, sex, albumin-bilirubin level, and platelet count to calculate the HCC risk. The aMAP score satisfactorily predicted the HCC risk in a cohort of 17,000 patients with viral and non-viral hepatitis from 11 global prospective studies and was excellent in discriminating and calibrating the 5-year HCC risk irrespective of the hepatitis etiology [141].

International society guidelines frequently quote an annual HCC risk threshold of ≥1.5% above which HCC screening is “recommended” and considered cost-effective [137,138]. However, the treatment success of anti-HCV therapy has improved since these cost-effectiveness analyses were performed. Newer cost-effectiveness analyses with a specific focus on post-SVR HCV patients indicates that surveillance could be cost-effective in patients with an annual risk of >1% [142,143].

## 20. Conclusions

Chronic infections with HBV or HCV are the dominant causes of HCC globally. The growing body of evidence on the direct and indirect hepatocarcinogenic effects of these viruses underscores the importance of viral eradication as a secondary prevention measure for HCC. Over the last years, a long list of retrospective and prospective studies has convincingly demonstrated the HCC-preventive effects of anti-viral therapy. As far as HBV-related carcinogenesis is concerned, there is overwhelming evidence for the positive impact of the pharmacological suppression of HBV on the risk of HCC. However, even patients who achieve cure following anti-HBV or anti-HCV therapy can still have a persistent residual HCC risk. In this respect, the implementation of dedicated HCC surveillance programs for those patients with the highest HCC risk remains essential. Research continues to be invested in developing novel anti-HBV or anti-HCV therapeutic modalities that could eliminate the HCC risk and develop a predictive model of HCC risk and the best surveillance strategies.

## Figures and Tables

**Table 1:** Models that could predict HCC development in patients with HBV.

	Chinese University Model [40]	Guide with Age, Gender, HBV DNA, Core Promoter Mutations, and Cirrhosis (GAG Model) [53]	Risk Estimation for HCC in CHB (REACH-B Model) [54]	Modified REACH-B Model [57]	Liver Stiffness Measurement Model [47]	Score Based on Age, Gender, and Platelet Count for HCC in CHB [55]
** Items **	Age Albumin Bilirubin Cirrhosis HBV DNA level	Age Gender BCP mutation Cirrhosis HBV DNA level	Age Gender ALT level HBeAg status HBV DNA level	Age Gender ALT level HBeAg status Liver stiffness value	Age Albumin HBV DNA level Liver stiffness value	Age Gender Platelet level
** Negative Predictive Value **	97% at 10 years	99% at 10 years	98% at 10 years	96.8% at 5 years	99.4% at 10 years	100% at 5 years

BCP: basal core promoter; ALT, alanine aminotransferase.

**Table 2:** Selection of retrospective and prospective studies evaluating the incidence of de novo HCC in DAA-treated HCV patients (adapted from Muzica et al.) [116].

Reference	Patient Population	Follow-Up	De Novo HCC Incidence in DAA-Treated Patients
**Retrospective Studies**			
Conti et al. [108]	*n* = 285; cirrhotic, DAA-treated	Mean 5.6 months	3.26%
Ravi et al. [110]	*n* = 66; cirrhotic, DAA-treated	6 months	6-month rate: 9.1%
Cardoso et al. [109]	*n* = 240; cirrhotic DAA-treated	Median 12 months	1-year rate: 7.4%
Singer et al. [111]	Chronic HCV, DAA-treated (*n* = 30,183), IFN-treated (*n* = 12,948), or untreated (*n* = 13,7502)	Mean 1.05 years	1.18 per 100 person-years
Nahon et al. [113]	Compensated cirrhotic; DAA-treated (*n* = 336), IFN-treated with SVR (*n* = 495), or IFN-treated without SVR (*n* = 439)	Median 21.2 months	2.6 per 100 person-years
Ioannou et al. [117]	DAA-treated (*n* = 21948), IFN-treated (*n* = 35871), DAA + IFN treated (*n* = 4535)	Mean 6.1 years	1.32 per 100 person-years
Kanwal et al. [13]	*n* = 22,500; DAA-treated	Mean 1.02 years	1.18 per 100 person-years
Kanwal et al. [118]	*n* = 18,076; DAA-treated, SVR	Mean 2.9 years	3-year rate: 3%
Janjua et al. [112]	IFN-treated (*n* = 8871), DAA-treated (*n* = 3905), SVR	Median 1.0 year	6.9 per 1000 person-years
Tani et al. [119]	DAA-treated (*n* = 1088)	Median 13.8 months	3-year rate: 3.71%
Watanabe et al. [120]	DAA-treated (*n* = 1438)	Median 803 days	3.82%
**Prospective Studies**			
Cheung et al. [121]	DAA-treated (*n* = 406), untreated (*n* = 261); decompensated cirrhosis	Median 18 months	4%
Mettke et al. [122]	DAA-treated (*n* = 158), untreated (*n* = 184)	Median 440 days	2.9 per 100 person-years
Carrat et al. [114]	DAA-treated (*n* = 7344), untreated (*n* = 2552)	Median 33.4 months	1.4 per 100 person-years
Poordad et al. [123]	DAA-treated (*n* = 2211)	156 weeks from end of treatment	1.4%
Piñero et al. [115]	DAA-treated (*n* = 1.017)	Median 16 months	Cumulative incidence 0.04 at 24 months
Sangiovanni et al. [124]	DAA-treated (*n* = 1285)	Mean 17 months	3.1 per 100 person-years
Romano et al. [125]	DAA-treated (*n* = 3917)	Median 523 days	0.97 per 100 person-years

## Data Availability

Not applicable.
